# Consciousness and cortical responsiveness: a within-state study during non-rapid eye movement sleep

**DOI:** 10.1038/srep30932

**Published:** 2016-08-05

**Authors:** Jaakko O. Nieminen, Olivia Gosseries, Marcello Massimini, Elyana Saad, Andrew D. Sheldon, Melanie Boly, Francesca Siclari, Bradley R. Postle, Giulio Tononi

**Affiliations:** 1 Department of Psychiatry, University of Wisconsin, Madison, WI, USA; 2 Department of Neuroscience and Biomedical Engineering, Aalto University School of Science, Espoo, Finland; 3 Department of Psychology, University of Wisconsin, Madison, WI, USA; 4 Coma Science Group, GIGA-Research & Neurology Department, University and University Hospital of Liege, Liege, Belgium; 5 Department of Clinical Sciences “Luigi Sacco”, Università degli Studi di Milano, Milan, Italy; 6 Don C. Gnocchi Foundation IRCCS, Milan, Italy; 7 Department of Neurology, University of Wisconsin, Madison, WI, USA; 8 Centre for Investigation and Research on Sleep, Centre Hospitalier Universitaire Vaudois and University of Lausanne, Lausanne, Switzerland

## Abstract

When subjects become unconscious, there is a characteristic change in the way the cerebral cortex responds to perturbations, as can be assessed using transcranial magnetic stimulation and electroencephalography (TMS–EEG). For instance, compared to wakefulness, during non-rapid eye movement (NREM) sleep TMS elicits a larger positive–negative wave, fewer phase-locked oscillations, and an overall simpler response. However, many physiological variables also change when subjects go from wake to sleep, anesthesia, or coma. To avoid these confounding factors, we focused on NREM sleep only and measured TMS-evoked EEG responses before awakening the subjects and asking them if they had been conscious (dreaming) or not. As shown here, when subjects reported no conscious experience upon awakening, TMS evoked a larger negative deflection and a shorter phase-locked response compared to when they reported a dream. Moreover, the amplitude of the negative deflection—a hallmark of neuronal bistability according to intracranial studies—was inversely correlated with the length of the dream report (i.e., total word count). These findings suggest that variations in the level of consciousness within the same physiological state are associated with changes in the underlying bistability in cortical circuits.

Understanding the neural substrates of conscious experience is a long-standing challenge^1^. Recently, studies using navigated transcranial magnetic stimulation coupled with high-density electroencephalography (TMS–EEG) have uncovered some tantalizing hints. These studies have revealed clear-cut differences between conscious and unconscious conditions during wakefulness, sleep, anesthesia, and severe brain injury^2^. When subjects are conscious (i.e., they have any kind of experience, like seeing an image or having a thought), TMS triggers a complex response made of recurrent waves of phase-locked activity. This is the case whether they are in a normal waking state (eyes open or closed)^3^,^4^ or are asleep but dreaming, as is usually the case during rapid eye movement (REM) sleep^5^; whether they are anesthetized with ketamine and unresponsive but report dreams upon awakening^6^; whether they have severe brain injuries but show signs of being in a minimally conscious state or locked-in syndrome^7^,^8^. By contrast, when subjects are considered unconscious and do not report any dreams, like typically in non-REM (NREM) sleep early in the night, in propofol-, xenon-, or midazolam-induced anesthesia, and in the unresponsive wakefulness syndrome/vegetative state, TMS triggers a slow-wave-like response that is larger but simpler^6^,^7^,^9^,^10^,^11^.

Recent experiments employing intracerebral electrical stimulations and recordings in humans indicate that during early NREM sleep the slow-wave-like response evoked by a cortical perturbation is associated with the occurrence of a cortical down-state^12^. Interestingly, after the down-state cortical activity resumes to wakefulness-like levels, but the phase-locking to the stimulus is lost, indicative of a break in the cause–effect chain. Overall, these findings suggest that bistability—the tendency of cortical neurons to fall into a down-state after an initial activation—may play a major role in reducing the ability of cortical neurons to engage in reciprocal causal interactions and, thus, in the brain’s capacity for consciousness^13^,^14^.

None of the aforementioned studies, however, have investigated whether the reactivity of cortical circuits changes when the level of consciousness varies *within* the same physiologically-categorized state, such as NREM sleep. This investigation is challenging but crucial if one wants to dissociate the neural correlates of consciousness *per se* from several other variables that may be changing across different states of vigilance. For instance, cognitive factors such as environment monitoring and readiness for task performance are present during wake but absent in sleep. Similarly, physiological factors such as the global level of brain activity or the level of neuromodulators differ in wake and sleep. It is well documented that subjects report unambiguous dream-like experiences when awakened from NREM sleep more than half of the time, and a third of the time an unambiguous lack of consciousness^15^,^16^,^17^,^18^,^19^. In a recent study, we showed that when subjects report a conscious experience after being awakened from NREM sleep, the preceding EEG is locally activated over a parieto-occipital area, while other brain areas exhibit slow-wave (delta) activity. On the other hand, if the same parieto-occipital region shows delta activity, subjects report no conscious experience before awakening^20^. We hypothesized that targeting the posterior parietal cortex with TMS within the same physiological state—NREM sleep—should reveal changes in cortical reactivity and underlying cortical bistability, depending on the presence or absence of a conscious experience.

We recorded TMS-evoked responses in six healthy participants using a 60-channel TMS-compatible EEG amplifier during NREM sleep in the course of 29 experimental study nights (four to five nights per subject). After observing a minimum of three minutes of NREM sleep, we applied single-pulse TMS to the medial superior posterior parietal cortex by means of magnetic resonance image (MRI)-guided estimation of the electric field induced in the cortex. The stimulation and recording procedures were similar to our previous experiments^7^,^10^ (see also Methods). After each TMS session, participants were awakened from NREM sleep by a sound and asked about the presence of conscious experience (“Tell me everything that was going through your mind before the alarm sound”). The participants’ responses were separated into three cases: no conscious experience (NCE), conscious experience without recall of content, or conscious experience with recall of content (a dream report). In the first two cases, subjects were further asked whether they were sure about their answer, after which they were told to go back to sleep. In the latter case, a full dream report was obtained and additional structured questions assessing the content of their experience were administered (not discussed here). Participants were awakened up to 16 times per night. This serial-awakening paradigm has been shown to be a valuable tool for studying sleep consciousness^15^,^21^,^22^. For comparison, the same target was also stimulated with TMS during wakefulness (during the first day session and in the evenings before the subjects went to sleep).

## Results

Of the 244 questionings after NREM sleep, 21 questionings were excluded from the analysis because the participants were too confused to understand/answer the questions, or because they awakened during the administration of the TMS. Twenty-nine additional questionings were rejected because the subjects were not sure about their answers. Finally, seven recordings had to be excluded due to low-quality signals in a large number of channels. Out of the 187 remaining questionings, 70 (37%) were associated with conscious experiences with recall, 39 (21%) with conscious experiences without recall, and 78 (42%) with NCE. These proportions are in line with previous studies^15^,^23^,^24^,^25^. Note that the reported conscious experiences were usually shorter (e.g., “I was dreaming about eating cookies, there was frosting on them” or “I was talking to a sports coach”) and less vivid than reports typically given after awakenings from REM sleep^15^. In the subsequent analyses, conscious experiences with and without recall of content were considered together (CE), as our aim was to investigate the presence of consciousness, regardless of whether the participants were able to recall the content of the experience or not.

**Figure 1:**
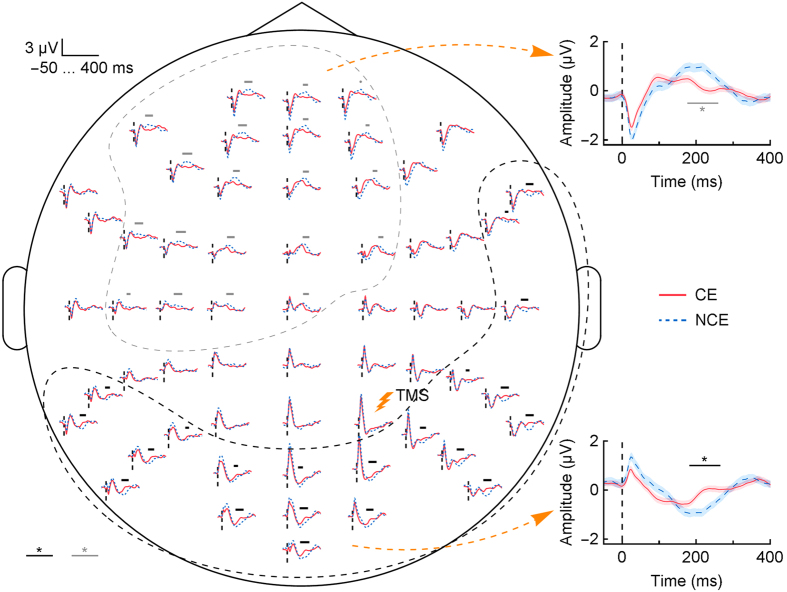
TMS-evoked EEG responses. The responses averaged across participants and plotted over a scalp map of the electrode positions. The black and gray bars indicate the channels and time points belonging to two clusters with significant differences between the CE (red) and NCE (blue) conditions [*P* = 0.042 for the anterior cluster and *P* = 0.031 for the posterior cluster]. The insets show the responses further averaged across the channels belonging to the clusters (indicated by the dashed lines); the horizontal bars mark significant differences between the CE and NCE conditions. The shaded areas represent mean ± standard error. The vertical dashed lines depict the moment of TMS. *Indicates *P* < 0.05. CE = conscious experience, NCE = no conscious experience.

Artifact-free filtered TMS–EEG data (1.5–45 Hz) of the wakefulness session and of the last 30 seconds before the awakenings were averaged across all sessions and subjects (see Methods for more details). We considered the last 30 seconds of the NREM data in order to strike a balance between two competing factors. First, we wanted to maximize the temporal proximity to the awakenings, when brain state and reports were most likely in synch^20^. Second, we needed sufficient time to accumulate enough trials (TMS pulses) to distinguish between the CE and NCE conditions using cluster-based permutation statistics (see Methods). We had a total of 1187 and 923 trials for the CE and NCE conditions, respectively. As previously reported, during wakefulness TMS triggered a complex response made of recurrent waves of activity, whereas during NREM sleep TMS induced a low-frequency wave that was larger and simpler (see Fig. 1 for NREM and Supplementary Fig. S3 for wakefulness results)^3^. Crucially, a clear difference was measured within NREM sleep when participants reported a conscious experience (CE) compared to when they reported its absence (NCE) (Fig. 1). The negative peak amplitude of the TMS-evoked response was larger, specifically at 200 ms after TMS, when subjects reported no conscious experience (insets of Fig. 1; see Supplementary Fig. S2a for individual data, *P* = 0.015 in the posterior cluster and *P* = 0.018 in the anterior cluster identified by a scalp-level group analysis). On the other hand, when subjects reported a conscious experience, this component was reduced and the TMS-evoked response contained more deflections and had smaller amplitude. The two clusters with opposite signal polarity likely reflect the presence of a common neuronal source, the strength of this source being linked to the level of consciousness.

**Figure 2:**
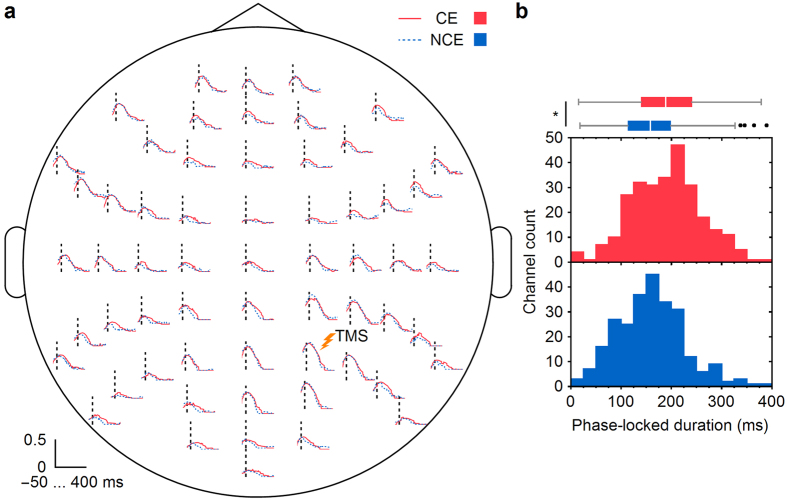
Phase-locking of the TMS-evoked EEG responses. (**a**) PLF for the CE (red) and NCE (blue) conditions averaged across participants (non-significant post-TMS points put to zero) and presented over a scalp map of the electrode positions. The vertical dashed lines indicate the moment of TMS. (**b**) Histograms of the phase-locked durations of the TMS-evoked responses in the 60 EEG channels of the six participants for the CE and NCE conditions. The boxplot illustrates the difference in the median durations between the CE and NCE conditions (see Methods). *Indicates *P* < 0.05. CE = conscious experience, NCE = no conscious experience.

Then, in order to investigate possible changes in the ability of cortical circuits to sustain causal interactions we calculated the instantaneous phase-locking factor (PLF). PLF quantifies (on a scale from 0 to 1) the ability of TMS to affect the phase of the underlying oscillations across trials, from the Hilbert transform of the data^26^. We calculated PLF for each channel of the individual participants and for three classical frequency bands (alpha, beta, and gamma) as well as for the spindle band. We set to zero all the non-significant post-TMS time points and took the first time point where PLF went back to zero value to determine the duration of the phase-locked response (see Methods). We observed an increase in the phase-locked duration when the participants did report conscious experiences, relative to when they did not, in the alpha band (8–13 Hz, *P* = 0.028; Fig. 2; see Supplementary Fig. S2b for the individual data), but not in the beta (13–30 Hz, *P* = 0.25), gamma (30–45 Hz, *P* = 0.51), or spindle (12–15 Hz, *P* = 0.80) ranges (one-tailed permutation tests, see Methods). Median durations were 190 ms for CE and 159 ms for NCE in the alpha band, 62 ms for CE and 59 ms for NCE in the beta band, 59 ms for CE and 57 ms for NCE in the gamma band and 153 ms for CE and 161 ms for NCE in the spindle range. The phase-locked duration during wakefulness was even longer than in the CE condition (226 ms in the alpha band, Supplementary Fig. S2).

**Figure 3:**
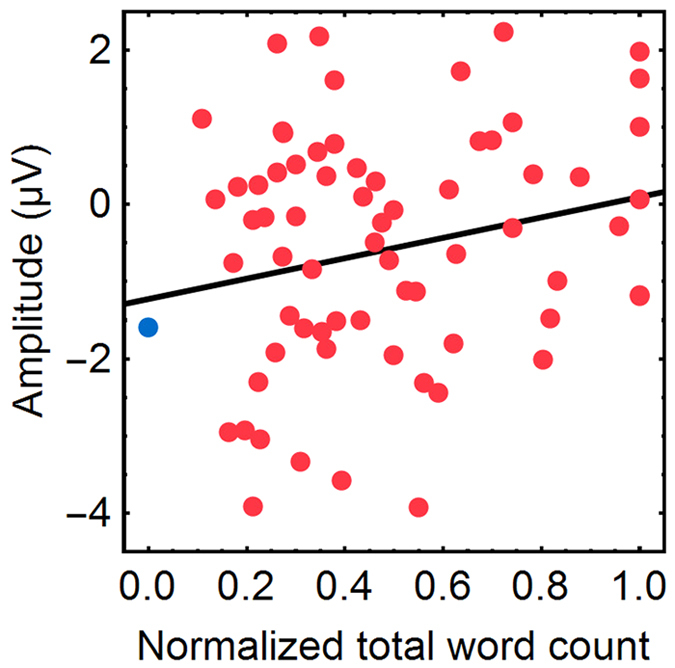
Correlation between the length of the conscious experience and the amplitude of the TMS-evoked response for all participants. The red dots represent the signal amplitude of the TMS-evoked response and the normalized total word count of individual awakenings. The line shows a linear fit to the data with *r* = 0.21 and *P* = 0.039. The blue circle represents the average amplitude of the data associated with no conscious experience.

We then examined the length of the dream reported upon awakening and found that it was positively correlated with the amplitude of the TMS-evoked deflection occurring at around 200 ms after TMS (Fig. 3) [*r* = 0.21, *P* = 0.039, one-tailed permutation test]. In other words, when the description of the conscious experience was short, the deflection of the TMS-evoked response was large. The length of the report was assessed by the total number of words the participants used to report their dreams divided by the maximum number of words the subject used. The amplitude of the TMS-evoked deflection was measured by subtracting the signal amplitude of the anterior cluster from that of the posterior cluster (see Methods and Supplementary Fig. S2c for the individual data). Consistent with the fit in Fig. 3, the amplitude difference between the clusters for the NCE data (0 words) was −1.6 ± 0.3 μV (mean ± standard error).

**Figure 4:**
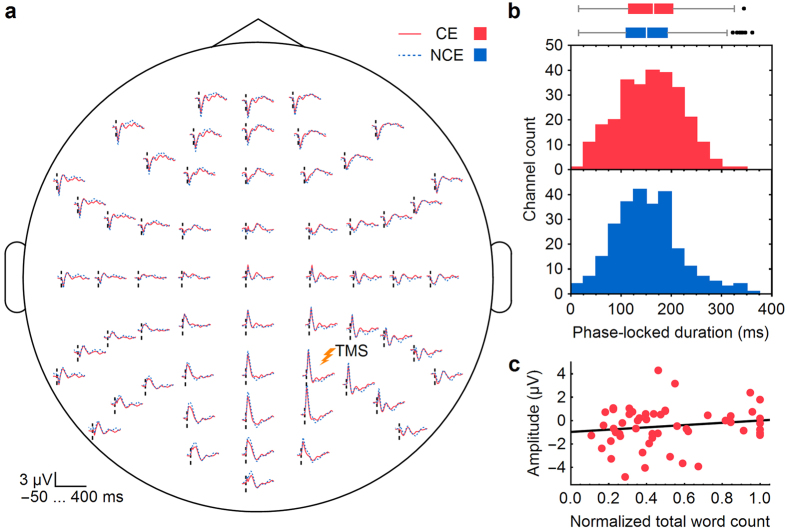
Control results for the trials occurring 2–2.5 minutes before the awakenings. Differences between the CE and NCE conditions (**a**,**b**) and the correlation (**c**) are non-significant [*P* > 0.05]. (**a**) TMS-evoked EEG responses averaged across subjects and plotted over a scalp map of the electrode positions. The vertical dashed lines depict the moment of TMS. (**b**) Histograms of the phase-locked durations of the TMS-evoked responses in the 60 EEG channels for the six participants. The boxplot illustrates the durations for the CE and NCE conditions [*P* = 0.34]. (**c**) Correlation between the amplitude of the TMS-evoked response at 2–2.5 minutes before the awakenings and the length of the conscious experience [*r* = 0.15, *P* = 0.13]. The red dots represents the signal amplitude of the TMS-evoked responses and the normalized total word count of individual awakenings. The line represents a linear fit to the data. CE = conscious experience, NCE = no conscious experience.

Finally, we conducted a control analysis on the trials occurring 2–2.5 minutes before the awakenings and found no differences for the evoked-response, PLF, and correlation analyses between the CE and NCE conditions (Fig. 4 and Methods). This supports the hypothesis that the CE and NCE reports captured changes in brain states during the last moments before the awakenings.

## Discussion

In summary, TMS applied to a posterior cortical region shortly before subjects are awakened from NREM sleep induces a different EEG response when subjects do or do not report having been conscious before the awakenings. Specifically, when subjects said they were not conscious, the EEG response was larger—similar to a larger NREM-sleep slow-wave—and the period of phase-locking was shorter. Importantly, this kind of response is characteristic of cortical networks when they are in a condition of bistability between depolarized up-states and hyperpolarized down-states^27^. At the single-cell level, bistability means that any input quickly triggers a stereotypical neuronal down-state, after which neurons enter an up-state and activity resumes stochastically, and is thought to be due primarily to depolarization-dependent potassium currents and short-term synaptic depression prevalent in NREM sleep^28^. Indeed, the spontaneous slow waves that characterize NREM sleep can be considered as a prime expression of neuronal bistability. In line with the present findings, a recent study in patients undergoing intracerebral recordings as part of a pre-surgical evaluation for refractory seizures showed that, while during wakefulness intracranial electrical stimulation triggers a chain of phase-locked activations in its cortical targets, during NREM sleep the same input often induces a stereotypical slow wave associated with a cortical down-state (suppression of power at frequencies above 20 Hz)^12^.

Cortical bistability, as reflected in the loss of phase-locking to a stimulus, leads to a breakdown in the ability of the cortex to integrate information, which is thought to be essential for being conscious^1^,^29^,^30^. More specifically, the decrease of phase-locked activity in the alpha band suggests that bistability may interfere primarily with feedback processes among distributed cortical areas, which has also been linked to consciousness^31^,^32^,^33^. The inverse relationship between bistability and consciousness is further supported by our observation that the amplitude of the TMS-evoked potential (indicative of greater bistability) was correlated with the length of the report (shorter conscious experiences). Overall, our findings suggest that local changes in the bistability of posterior cortical networks, as revealed by TMS–EEG responses, can faithfully reflect variations in the level of consciousness. Moreover, since changes in TMS–EEG responses predict changes in consciousness within the same physiological state—NREM sleep—these findings highlight the importance of bistability while ruling out possible confounding factors due to physiological state changes. However, there is still a caveat, as a truly homogenous state is an abstraction: there is still some variability within NREM sleep and, in fact, one can categorize NREM sleep based on type-1 and type-2 waves^34^ as well as the predominant location of the wave (front versus back)^20^, not to mention the different ways these slow waves can travel^35^. Future studies should next look at how TMS–EEG responses and dream reports in NREM sleep depend on the pre-stimulus endogenous oscillatory pattern, as the amplitude of the TMS-evoked response has been shown to correlate with the preceding slow-wave amplitude and specific timing of TMS (with respect to peaks and troughs)^36^.

## Methods

### Participants

Eleven healthy subjects (2 females, age 26.1 ± 3.5 years, 19–30 years (mean ± standard deviation, range)) were recruited for the study and received monetary compensation for their participation. Prior to the experiment, participants were screened for neurological, psychiatric (MINI 5.0.0), mood (HAM-D), and sleep disorders using a structured interview. Participants had no contraindications for TMS (e.g., history of seizures) or MRI (e.g., metal implant), and none of them was on psychotropic medication. They had good sleep quality as assessed by the Pittsburgh Sleep Quality Index^37^. We discarded five participants because one displayed TMS-evoked responses with a very low signal-to-noise ratio during the first wakefulness session, two were unable to sleep in the laboratory environment (one night and two nights, respectively), one perceived phosphenes during TMS (and interrupted the study before the last two nights), and the last subject presented auditory evoked responses due to the TMS coil click despite the noise masking. The data presented here are therefore from six right-handed participants who completed the study (5 males, age 23.7 ± 3.2 years). Note that for subject 6, we increased the TMS intensity (from 100 to 125 V/m) after the fourth night, as the NCE and CE responses differed from those of the other subjects (which could have been due to a low stimulation intensity that could not trigger slow-wave-like responses—see supplementary discussion about individual data). Thus, here we report the results using the data of the last four nights that were acquired with the higher intensity. Written informed consent was obtained from each participant. The study was approved by the University of Wisconsin Human Subjects Committee and was carried out in accordance with the Declaration of Helsinki^38^.

### Data acquisition

Participants were first invited to the laboratory to perform the clinical screening, an MRI acquisition, and a TMS–EEG session (during wakefulness and a nap) to ensure the subject’s eligibility and good TMS–EEG responses. For the two weeks before the first night at the laboratory, participants were asked to fill out a questionnaire (see below) every morning to become familiar with reporting conscious experience upon awakening. Five overnight recordings were scheduled for each participant in the form of three consecutive nights followed by two consecutive nights (1 + 3 nights for subject 6). In total, we performed 46 overnight experiments (29 nights reported here) and 13 wakefulness sessions during daytime. During the experiments, participants were lying on their back on a comfortable reclining chair, and depending on the participant’s sleep schedule, continuous EEG recordings were started between 10 p.m. and 1 a.m. and ended between 7 and 10 a.m.

Participants were awakened during the night (after each TMS session, see below) by a computerized alarm sound lasting 1.5 seconds. They were instructed to signal that they had heard the sound, and we waited 10 seconds before asking the questions so that they could think about what to answer. We also requested from our participants that they inform us if they woke up during the administration of the TMS. Interviews were then conducted at the bedside with a structured questionnaire, and answers were audiotaped and later transcribed. The first question was “Tell me everything that was going through your mind before the alarm sound/you woke up”. Participants were instructed to report whether they had a conscious experience by delivering a dream report, report that they had had a conscious experience but did not remember its content, or report that they had experienced nothing. In the last two cases, they were further asked whether they were sure about their response. We also asked each subject to report their subjective level of wakefulness. When subjects reported a conscious experience with recall of content, other questions concerning the content of their experience were also asked, such as the degree of thinking and perceiving (not discussed in the present article).

Electrical brain activity was recorded using a 60-channel TMS-compatible EEG amplifier (Nexstim eXimia, Nexstim Plc, Finland) with a sample-and-hold circuit, which prevents the amplifier from saturation. EEG was referenced to an additional channel on the forehead, filtered (0.1–350 Hz), and sampled at 1450 Hz. Two additional electrodes were used to record the electrooculogram (Nexstim eXimia, Nexstim Plc, Finland) and four sensors for the electromyogram were placed on the chin (BrainAmp, Brain Products GmbH, Germany, 5-kHz sampling frequency). We prepared the EEG channels to each have an impedance of less than 5 kΩ; throughout the night, we monitored the impedance of each channel and adjusted it when necessary.

NREM sleep was scored online over 30-second epochs according to the American Academy of Sleep Medicine (AASM) Scoring Manual^39^. After observing a minimum of three minutes of NREM sleep (stages 2 and 3), we applied single-pulse TMS to the medial superior parietal cortex (superior parietal lobule and precuneus, see Supplementary Table S1). We chose to target this area for several reasons. First, previous studies have identified it as a hub in brain networks, it is the area most often involved in neural correlates of consciousness, and it includes extensive corticocortical connections^20^,^40^,^41^. Second, it can be conveniently stimulated without eliciting muscle artifacts^42^. Third, it is easily accessible while participants are asleep. Finally, its TMS response is well-documented^4^. The precise cortical TMS targets (Supplementary Table S1) that provided good TMS-evoked EEG responses were identified individually for each subject during the daytime session (two sessions for subjects 2 and 3) and marked on the T1 MR images of the participants’ heads acquired with a 3-T MRI scanner (Discovery MR750 3.0T, GE Healthcare, UK). The same target was stimulated in all night sessions. As in our previous studies^3^,^7^,^43^, we employed a Navigated Brain Stimulation system (eXimia NBS, Nexstim Plc, Finland) to ensure precise and reproducible stimulation. Specifically, a stereotactic infrared camera tracked the position and orientation of the coil with respect to the subject’s head. The head-tracker goggles were firmly taped on the EEG cap and the co-registration for the navigation was performed several times during the night to ensure exact targeting. The location of the maximum electric field induced by TMS in the cortex was on the convexity of the targeted gyrus with the induced current perpendicular to it and oriented so that the predominant stimulation direction was in the posterior–anterior direction. Biphasic TMS pulses (between 10 and 284 per session) were delivered using a figure-of-eight coil (Focal BiPulse, Nexstim Plc, Finland)^44^ at random intervals (2–2.3 seconds), and the maximum electric field at the cortical target was between 100 and 130 V/m (70–83% of the maximum stimulator output, MSO). The stimulation parameters were in accordance with published TMS guidelines^45^. To prevent the participants from waking up, we started stimulating at 30% (40% for subject 6) of MSO and increased the intensity by 10%-MSO steps every two stimulations, until the chosen intensity was reached. The stimulation was discontinued at any noticeable sign of arousal. In 78 sessions (44 CE and 34 NCE), subjects woke up during the stimulation, and the questions were asked without playing the alarm sound. To avoid auditory evoked potentials due to the TMS coil click, participants wore earphones with noise masking, and a thin foam pad was placed between the scalp and the coil^11^,^46^. Between the TMS sessions, the coil was cooled using ice packs. At the end of the experiments, the electrode positions and scalp landmarks (nasion, left and right tragus) were digitized. The digitization data were used to ensure the alignment of the EEG cap across multiple nights. In total, we performed 244 sessions in NREM sleep (up to 16 sessions per night; 40, 40, 26, 44, 49, and 45 sessions for subjects 1–6, respectively). We also applied TMS during REM sleep and NREM sleep stage 1 (data not shown), as well as during wakefulness before participants went to sleep.

### Data pre-processing

Data analysis was performed using Matlab (The MathWorks, Inc., MA, USA) and Mathematica (Wolfram Research, Inc., IL, USA) scripts generated specifically for this study, unless otherwise mentioned. The recorded EEG data were sleep scored offline according to the AASM manual^39^ using EEGLAB^47^ to ensure that all considered TMS sessions were recorded during NREM sleep (stages 2 and 3) with at least three preceding minutes of sleep without stage transitions. For each session, trials with artifacts or brief arousals were manually rejected using The SiSyPhus Project Matlab program (University of Milan, Italy). Bad channels were detected visually and later interpolated^48^; seven sessions (1 CE and 6 NCE sessions) that had over seven bad channels were excluded from the analysis. To avoid contamination from TMS-related artifacts present in some trials, the first 15 ms of the data post-TMS were removed and linearly interpolated. The data were band-pass filtered between 1.5 and 45 Hz by applying a second-order Butterworth filter in the forward and backward directions and down-sampled to 362.5 Hz. The data were then epoched with respect to the TMS pulses, baseline-corrected using a 400-ms-long baseline interval, and average-referenced. Note that the data of subjects 1 and 4 were flipped with respect to the midline electrodes, as they were stimulated on the left hemisphere as opposed to the others. In these subjects, we chose to target the left hemisphere because, during the daytime session, it provided better TMS-evoked EEG responses (lower artifact level, larger response) than sites in the right hemisphere.

### Evoked-response analysis

For both the CE and NCE conditions, TMS-evoked responses were averaged over the last 30 seconds before the awakenings (Fig. 1, Supplementary Fig. S1, and Supplementary Fig. S2a). We had in total 1187 and 923 trials for the CE (only including the reports of conscious experiences with and without the recall of content of which subjects were sure; 774 trials for CE with recall of content and 413 trials for CE without recall of content) and NCE (only including the answers of which subjects were sure) conditions, respectively, with maximum 14 trials per session. The number of trials for each subject is shown in Supplementary Table S2. We also did the same analyses separating NREM sleep into stages 2 and 3, which gave results similar to those reported in the present article.

The statistical significance of the difference between the TMS-evoked responses in the CE and NCE conditions was assessed by cluster-based permutation statistics^49^. Specifically, we computed the *t*-statistics separately for each channel and time point (15–400 ms after TMS). We then thresholded the data by discarding the values corresponding to *P*-values above 0.05. Next, we computed cluster statistics by summing up the *t*-statistics of the neighboring data points (neighbors in time and in the channel space; the discarded points defined the cluster borders). By these means, we found the channels and samples belonging to several candidate clusters. To determine the significance of the clusters, we pooled the CE and NCE data, drew 10,000 random permutations, and computed the cluster statistics for these randomized datasets as the maximum cluster value of each permutation. Finally, the statistics of the candidate clusters were compared against the statistics distribution of the randomized data to obtain their two-tailed *P*-values, adjusted for multiple comparisons.

For the trials of the last 30 seconds, the aforementioned analysis identified two clusters (Fig. 1). For both of the clusters, we averaged the data across the channels belonging to the clusters at the single-trial level. The statistical significance of the differences between the CE and NCE conditions was again assessed by cluster-based permutation statistics as described above, except that now the neighbors occurred only in time.

Finally, note that the low number of trials associated with each awakening (up to 14 trials per session) was insufficient for computing the perturbational complexity index^2^.

### PLF analysis

In order to estimate the duration of the deterministic TMS-evoked response for both the CE and NCE conditions, we computed the phase-locking factor^12^,^26^ as a function of time (Fig. 2 and Supplementary Fig. S2b). PLF was assessed on a single-subject and single-channel level after band-pass filtering for the alpha (8–13 Hz), beta (13–30 Hz), gamma (30–45 Hz), and spindle (12–15 Hz) bands, respectively. The classical bands were selected because a recent study using intra-cerebral stimulations and EEG recordings showed that PLF in the alpha (8–13 Hz) and beta (13–30 Hz) bands was decreased in NREM sleep as compared to wakefulness, but similar in the gamma band (>30 Hz)^12^. Statistical differences from the baseline were calculated by assuming a Rayleigh distribution of the values of the baseline (from −400 to −100 ms) for each channel and subject. Then, PLF values below a statistical threshold (*α* < 0.05) were set to zero and the phase-locked duration was determined as the time of the first drop to zero. In order to quantify the difference between the CE and NCE conditions, we kept only those channels for each subject and condition in which the phase-locked duration was over 15 ms, thus effectively rejecting channels with a low signal-to-noise ratio. Then, we computed the median of the phase-locked duration across the channels and subjects. The statistical significance of the difference in the phase-locked durations between the CE and NCE conditions was assessed by permutation statistics. From the pooled CE and NCE data, we draw 10,000 randomized datasets and computed the phase-locked durations using the same method as with the original data. The original difference in the phase-locked duration was tested against the distribution of the permuted differences to obtain a one-tailed *P*-value. We used a one-tailed test because a previous study^12^ showed that the phase-locked duration decreased with a reduced level of consciousness.

To ensure that the difference in the phase-locked durations in the alpha band were not simply due to changes in the signal power, we controlled for it. Specifically, we computed the signal power for the studied frequency band as a root-mean-squared signal amplitude of the band-pass filtered single-trial data for the baseline and for 100-ms-long time windows starting every 50 ms (the first window at 15 ms after TMS). For each subject, channel, and time window, we then computed the uncorrected *P*-values for the null hypothesis that the signal power was the same as in the baseline. Of the 360 channels (60 channels × 6 subjects) only 14 had at least one time window with an uncorrected *P*-value below 0.05 that indicated potentially higher power at the alpha band for the CE as compared to the NCE condition. We then did another PLF analysis similar to the one described above in all aspects, except that we discarded those channels belonging to this group (5 of those were rejected also in the original analysis due to the 15-ms rejection threshold). This resulted in no change.

### Correlation analysis

We measured the correlation between the length of the subject’s report (the CE condition with recall of content) and the signal amplitude of the TMS-evoked EEG response (Fig. 3). The length of the report was calculated using the total word count (i.e., count of all words the subject produced to report their conscious experience). Note that the word count not only reflects the length of the experience but also its richness and vividness^50^. Because the number of words used to report the same experience is likely to vary according to the verbal tendencies of the subject, we normalized the total word count for each subject to their longest report. The amplitude of the deflection was calculated independently for each awakening session by averaging the trials over the last 30 seconds before the awakenings, averaging across the channels belonging to the posterior and anterior clusters shown in Fig. 1, respectively, and by calculating the mean signal amplitude for the clusters over the duration of the statistically significant difference indicated in the insets of Fig. 1 (181–255 ms and 186–261 ms for the anterior and posterior clusters, respectively). Finally, the value of the anterior cluster was subtracted from that of the posterior cluster. By this means, we obtained an objective selection of the channels and time points of interest. The two clusters with opposite signal polarity likely arise due to a common source between them; thus, the subtraction effectively averages the absolute signal amplitudes of all the channels that contain a statistically significant deflection associated with the same neuronal activity. For subject 6, we excluded one outlier point (32 words, 6.0 μV) from the analysis. The statistical significance of the correlation was assessed by a one-tailed permutation test, in which we created 10,000 random datasets by redefining word-count–amplitude pairs by permutations and computed the correlation for each of those datasets. Here, we used a one-tailed test because the correlation should be in line with the observed difference between the CE and NCE conditions (Fig. 1).

### Control analysis

We conducted the evoked-response, PLF, and correlation analyses also on the trials occurring 2–2.5 minutes before the awakenings and observed neither differences between the CE and NCE conditions nor a correlation (Fig. 4). For this time interval, we had 977 trials in the CE and 778 trials in the NCE condition (maximum 15 trials per session). The number of trials for each subject is shown in Supplementary Table S2. The number of channels included in the PLF analysis was 270 and 259 for the CE and NCE conditions, respectively. This latter analysis was performed as a sanity check to show that the difference between the CE and NCE conditions when using the data of the last 30 seconds before the awakenings was due to the reported presence or absence of a conscious experience. Indeed, no link should exist between the brain state and the subject’s reports when there is a long time gap between the awakenings and the utilized TMS-evoked EEG data.

### Wakefulness data

Finally, we analyzed data acquired during wakefulness while participants were fixating a cross on the wall, and compared it to the NREM data. We selected 200 artefact-free trials for each subject and averaged the evoked response across them (Supplementary Fig. S3a). We then computed the phase-locking of the responses in the alpha band (Supplementary Fig. S3b,c) in the same manner as for the CE and NCE conditions. The statistical significance of the difference in the phase-locked durations between wakefulness and the CE condition was assessed using permutation statistics, as previously done for the CE and NCE conditions.

## Additional Information

**How to cite this article**: Nieminen, J. O. *et al*. Consciousness and cortical responsiveness: a within-state study during non-rapid eye movement sleep. *Sci. Rep.* **6**, 30932; doi: 10.1038/srep30932 (2016).

## Supplementary Material

Supplementary Information
