# Supplementary material for: Consciousness and cortical responsiveness: a within-state study during non-rapid eye movement sleep
Source: Sci Rep. 2016 Aug 5;6:30932. doi: 10.1038/srep30932 (PMC4974655; doi:10.1038/srep30932)
Supplement: Supplementary Information [file srep30932-s1.pdf]

Supplementary information

Consciousness and cortical responsiveness: a within-state study during non-rapid eye movement sleep

Jaakko O. Nieminen[1,2]†, Olivia Gosseries[1,3,4]†, Marcello Massimini[5,6], Elyana Saad[1,3], Andrew D. Sheldon[1,3], Melanie Boly[1,7], Francesca Siclari[1,8], Bradley R. Postle[1,3], Giulio Tononi[1]*

[1]Department of Psychiatry, University of Wisconsin, Madison, WI, USA.

[2]Department of Neuroscience and Biomedical Engineering, Aalto University School of Science, Espoo, Finland.

[3]Department of Psychology, University of Wisconsin, Madison, WI, USA.

[4]Coma Science Group, GIGA-Research & Neurology Department, University and University Hospital of Liege, Liege, Belgium.

[5]Department of Clinical Sciences "Luigi Sacco", Università degli Studi di Milano, Milan, Italy.

[6]Don C. Gnocchi Foundation IRCCS, Milan, Italy.

[7]Department of Neurology, University of Wisconsin, Madison, WI, USA.

[8]Centre for Investigation and Research on Sleep, Centre Hospitalier Universitaire Vaudois and University of Lausanne, Lausanne, Switzerland.

†These authors contributed equally to this work.

*Correspondence to:

Prof. Giulio Tononi

Dept. of Psychiatry

University of Wisconsin

Madison, WI, USA

gtononi@wisc.edu

Supplementary discussion

Consciousness

In this article, we refer to consciousness in the sense of having a subjective experience, which may occur in the form of, e.g., an image, a thought, a scene, or an emotion. Dreaming during sleep is a valuable form of consciousness as it is an experience, though one not directly related to the current environment and with decreased volition, self-awareness, affect, and memory as compared to waking consciousness[1–3] (see below for more comparison between wakefulness and sleep consciousness). Because dreams in the general sense are usually associated with full-fledged, vivid, and long stories occurring in REM sleep[4], we here use the term "conscious experience", as we focus on NREM sleep (shorter reports as compared to REM sleep).

Because NREM experiences are reported while subjects are awake, one cannot however exclude that the inability to report conscious experiences (NCE) might not be only due to the absence of conscious experience *per se*, but also due to the inability to remember any conscious experience (i.e., a recall problem). An indirect way to look at this issue is to focus on the CE condition with and without recall of content. Our data show that the responses are actually similar among these two conditions (Supplementary Fig. S1), which suggests that if there were some conscious experiences in the NCE condition, it would only reduce the observed difference between the NCE and CE conditions. Another concern is how one could know to which temporal interval a report relates to, as in principle, the basis for the reported conscious experience could have happened minutes, seconds, or even milliseconds before the awakening. The reports by themselves do not come with a specific timestamp, and thus it remains uncertain when those experiences occurred. As such, the NCE versus CE contrast might underestimate the differences in the brain activity

between these two conditions. Finally, one could also argue that the conscious reports are only invented by the subjects upon awakening, but this has been addressed elsewhere (see ref. 5 for neural decoding of dream reports and ref. 6 for trustworthiness of first-person dream reports).

Behavioral observations

The serial-awakening paradigm was successful in our TMS–EEG setting. It was easy for participants to fall back asleep after waking up; specifically, in 90% of the cases, it took the subject less than 8 minutes to fall back asleep (i.e., reach stage-2 NREM sleep) after questioning. The answers given after waking up spontaneously (and hence without the alarm sound) were equally distributed among the CE and NCE conditions. This was comparable to the answers given when participants were awakened with the alarm; 44 of the 78 awakenings that occurred without the alarm and 66 of the 110 awakenings initiated with the alarm belonged to the CE condition. We had a high number of CE and NCE awakenings both in the NREM sleep stage 2 and 3 (68 CE and 35 NCE awakenings in stage 2; 41 CE and 43 NCE awakenings in stage 3), which suggests that the difference observed between the NCE and CE conditions is not due to the sleep stage. There was, however, high variability across participants on how often they recalled the content of their experience, as previously observed in other studies[7,8]. For example, subject 1 recalled the content of 15 out of 19 CEs, whereas subject 2 only recalled the content of 4 out of 19 CEs, which resulted in a low number of data points in Supplementary Fig. S2c.

Individual results

The overall results were globally consistent among the six participants. Five participants showed the typical TMS-evoked down-state response at around 200 ms in the NCE condition. However, subject 6 did not show such a response, instead showing an earlier down-state at 100 ms followed by an up-state at 200 ms (Supplementary Fig. S2a). Interestingly, there still seems to be a difference between the CE and NCE conditions at 200 ms, but it is characterized by the opposite pattern of that of the other participants. This discrepancy could be due to the fact that TMS did not trigger slow-wave-like responses in NREM sleep in that participant. Indeed, even after increasing the stimulation intensity, we did not observe typical NREM TMS-evoked responses. We therefore hypothesize that TMS has to induce slow-wave-like responses in order to be sensitive to changes in bistability in the brain and thereby obtain distinct responses between the CE and NCE conditions. The median phase-locked duration was longer in the CE than in the NCE condition for five participants (Supplementary Fig. S2b). The shorter phase-locked duration of subject 6 in the CE condition was likely due to the atypical TMS-evoked responses (Supplementary Fig. S2a). All but one subject showed a positive correlation between the total word count and the peak amplitude of the TMS-evoked response (Supplementary Fig. S2c), which is in line with the observed individual difference in the evoked responses between the CE and NCE conditions (Supplementary Fig. S2a). The Pearson's correlation coefficients for subjects 1–6 are 0.13, 0.91, 0.36, 0.43, 0.11, and −0.17, respectively. Note the opposite correlation for subject 6, which could again be explained by the absence of a typical down-state at 200 ms.

Wakefulness results

As previously observed, TMS in wakefulness induces sustained long-range and complex patterns of brain activation with spatiotemporally differentiated patterns associated with the natural frequency of the stimulated area and distant regions (Supplementary Fig. S3a)[9,10]. Consistently, the CE condition in NREM sleep is also associated with more deflections in the evoked responses (Fig. 1). The phase-locking of the response is also stronger in the CE compared to the NCE condition (190 vs. 159 ms), which is consistent with the longer-lasting phase-locking during wakefulness (226 ms for the alpha band, Supplementary Fig. S3c) as compared to the CE condition [$P = 0.03$, one-tailed permutation test]. Note that the phase-locking in wakefulness lasted longer also in the beta (128 ms) and gamma bands (87 ms) than in the CE condition [$P < 0.001$ for both bands, one-tailed permutation tests]. The phase-locking comparison at the alpha band may be affected by increased alpha-band power during wakefulness; when excluding the 167 channels with potentially higher power at this band for the awake as compared to the CE condition, the statistical test gave $P > 0.05$ (208 ms in wakefulness and 203 ms in the CE condition).

<u>**Supplementary references**</u>

1. Kahn, D. & Gover, T. Consciousness in dreams. *Int. Rev. Neurobiol.* **92,** 181–195 (2010).
2. Nir, Y. & Tononi, G. Dreaming and the brain: from phenomenology to neurophysiology. *Trends Cogn. Sci.* **14,** 88–100 (2010).
3. Domhoff, G. W. The neural substrate for dreaming: Is it a subsystem of the default network? *Conscious. Cogn.* **20,** 1163–1174 (2011).
4. Hobson, J. A., Pace-Schott, E. F. & Stickgold, R. Dreaming and the brain: Toward a cognitive neuroscience of conscious states. *Behav. Brain Sci*. **23,** 793–842 (2000).
5. Horikawa, T., Tamaki, M., Miyawaki, Y. & Kamitani, Y. Neural decoding of visual imagery during sleep. *Science* **340,** 639–642 (2013).
6. Windt, J. M. Reporting dream experience: Why (not) to be skeptical about dream reports. *Front. Hum. Neurosci.* **7,** 708 (2013).
7. Lewis, H. B., Goodenough, D. R., Shapiro, A. & Sleser, I. Individual differences in dream recall. *J. Abnorm. Psychol.* **71,** 52–59 (1966).
8. Marzano, C. *et al.* Recalling and forgetting dreams: Theta and alpha oscillations during sleep predict subsequent dream recall. *J. Neurosci.* **31,** 6674–6683 (2011).
9. Massimini, M. *et al.* Breakdown of cortical effective connectivity during sleep. *Science* **309,** 2228–2232 (2005).
10. Rosanova, M. *et al.* Natural frequencies of human corticothalamic circuits. *J. Neurosci*. **29,** 7679–7685 (2009).
11. Tzourio-Mazoyer, N. *et al.* Automated anatomical labeling of activations in SPM using a macroscopic anatomical parcellation of the MNI MRI single-subject brain. *Neuroimage* **15,** 273–289 (2002).

Supplementary figures

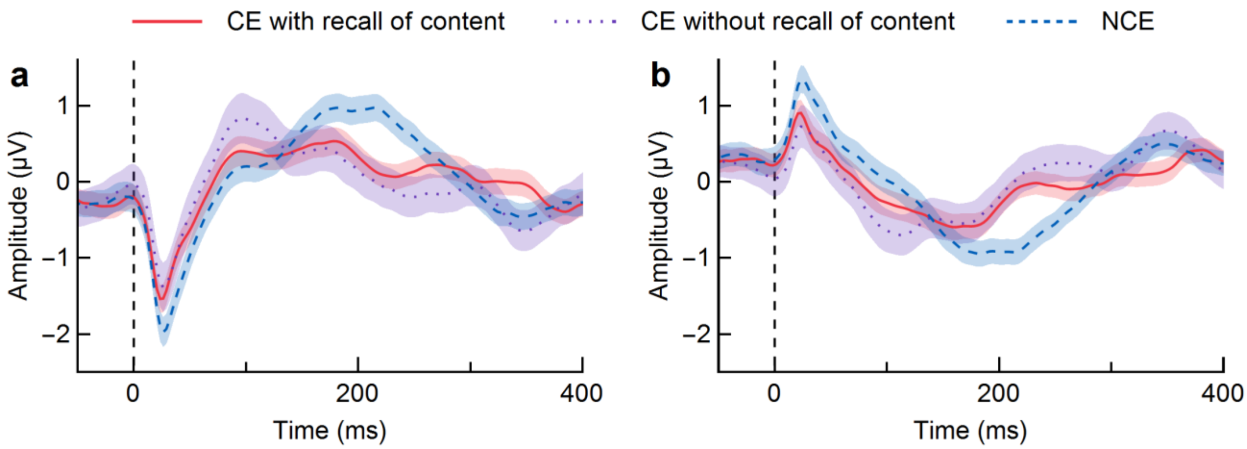

Supplementary Figure S1. Conscious experience (CE) with and without recall of content compared to no conscious experience (NCE). TMS-evoked responses averaged across subjects for the last 30 seconds before the awakenings. In (**a**) and (**b**), the signals are averaged across the channels belonging to the anterior and posterior clusters shown in Fig. 1, respectively. The shaded areas represent mean ± standard error. The vertical dashed lines depict the moment of TMS.

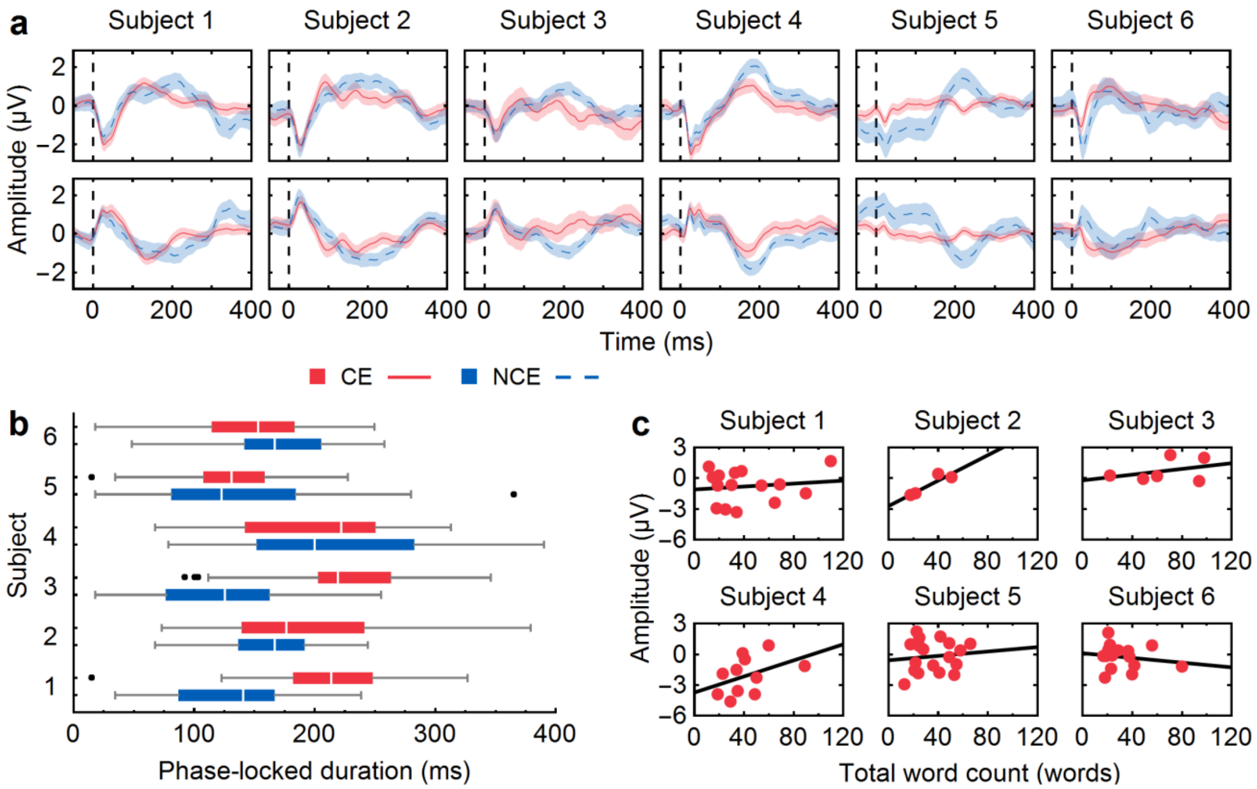

Supplementary Figure S2. Results for the individual subjects. (**a**) TMS-evoked responses for the six participants. The top and bottom rows show the signals averaged across the channels belonging to the anterior and posterior clusters shown in Fig. 1, respectively. The shaded areas represent mean ± standard error. The vertical dashed lines depict the moment of TMS. (**b**) Phase-locked durations of the TMS-evoked responses for each participant. The boxplot gives the duration distributions across the 60 EEG channels similar to Fig. 2b. (**c**) Correlation between the amplitude of the TMS-evoked EEG response and the length of the conscious experience for each participant. The red dots represents the signal amplitude and normalized total word count of individual awakenings. The lines present linear fits to the data.

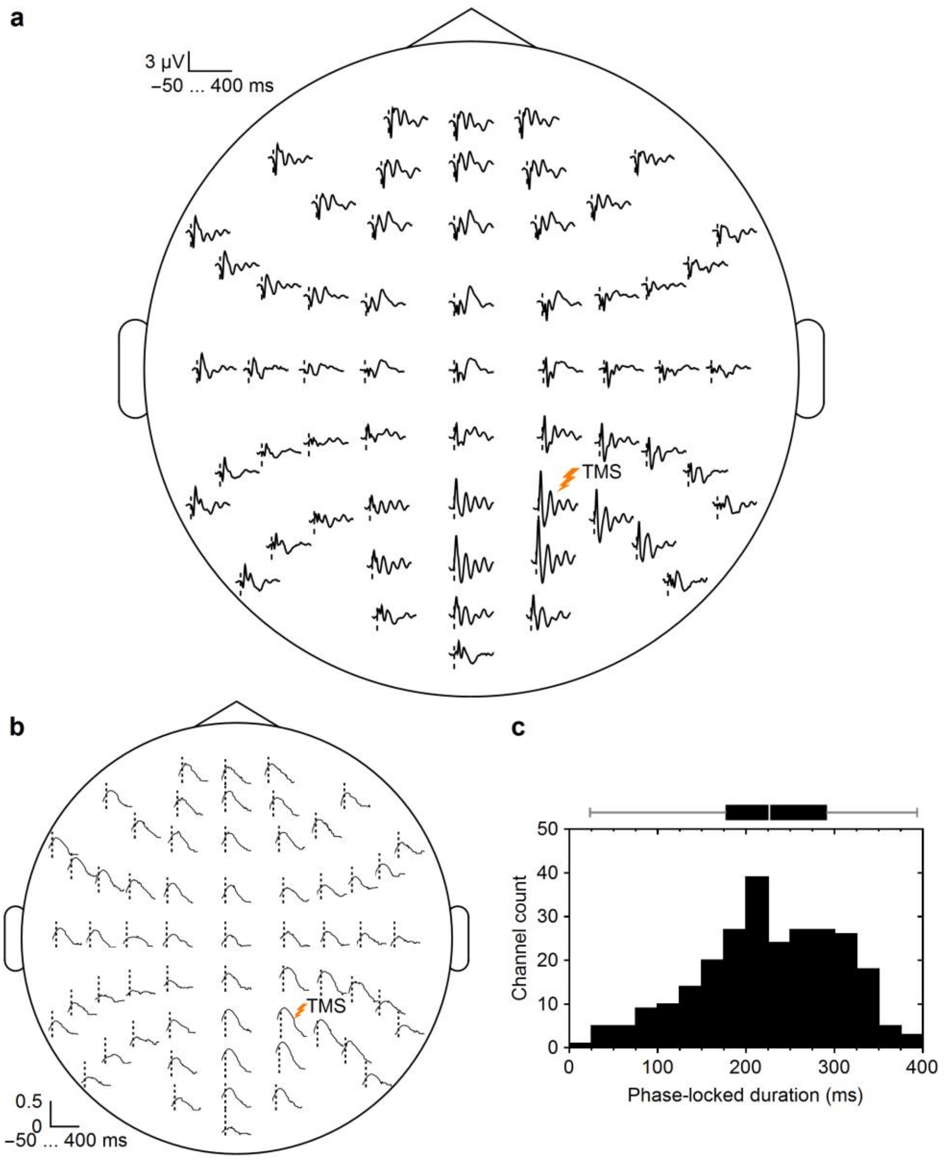

Supplementary Figure S3. Results in wakefulness. (**a**) TMS-evoked EEG responses averaged across participants and plotted over a scalp map of the electrode positions. The vertical dashed lines depict the moment of TMS. (**b**) PLF averaged across subjects (non-significant post-TMS points put to zero) and presented over a scalp map of the electrode positions. The vertical dashed lines indicate the moment of TMS. (**c**) Histogram of the phase-locked durations of the TMS-evoked responses in the 60 EEG channels of the six participants. The boxplot summarizes the distribution.

Supplementary tables

Supplementary Table S1. Stimulation sites. Montreal Neurological Institute (MNI) coordinates of the cortical TMS targets and the corresponding anatomical locations (according to the automatic anatomical labeling (AAL) atlas[11]) for the individual subjects.

Subject	*x*	*y*	*z*	Location
1	−5	−69	63	left precuneus
2	28	−68	55	right superior parietal lobule
3	18	−69	61	right superior parietal lobule
4	−11	−67	64	left precuneus
5	13	−72	60	right precuneus
6	12	−74	61	right precuneus

Supplementary Table S2. Number of trials per subject and condition.

Subject	Last 30 s		2–2.5 min	
	CE	NCE	CE	NCE
1	190	96	172	79
2	230	243	168	202
3	90	175	73	113
4	134	163	118	157
5	285	104	250	95
6	258	142	196	126
